# From poverty trap to ecosystem service curse

**DOI:** 10.1007/s11625-016-0370-8

**Published:** 2016-05-10

**Authors:** Jakub Kronenberg, Klaus Hubacek

**Affiliations:** 1 Department of International Economics, Faculty of Economics and Sociology, University of Lodz, P.O.W. 3/5, 90-255 Lodz, Poland; 2 Department of Geographical Sciences, University of Maryland, College Park, MD 20742 USA

**Keywords:** Poverty trap, Rigidity trap, Payments for ecosystem services, Institutions, Ecosystem services markets

## Abstract

In spite of broad and positive expectations, payments for ecosystem services (PES) can bring about unexpected and negative consequences, especially in terms of their impacts on the well-being of local communities dependent on ecosystems. Based on numerous observations of recurring problems with PES, we put forward an *ecosystem service curse* hypothesis (Kronenberg and Hubacek in Ecol Soc 18:art.10. doi:10.5751/ES-05240-180110, 2013), that points to counterintuitive negative development outcomes for countries and regions rich in ecosystem services. The social and economic problems that we have been able to depict in many PES schemes reflect the persistence of maladaptive states in pursuit of sustainability. Instead of providing an opportunity to break out of poverty, these problems reflect entrapment, which is most often related to poor quality of institutions. Here we highlight the linkages between the ecosystem service curse hypothesis and the dynamic system stability landscapes discussed in this special issue. Our article consists of three parts in which we: (1) present the original ecosystem service curse hypothesis; (2) link this hypothesis to the broader discussions relevant to sustainability science; and (3) highlight the context of traps on which this special feature focuses.

## Ecosystem service curse

Payments for ecosystem services (PES) are designed to reward those who maintain ecosystem quality (ecosystem service providers) through payments from those who benefit from the stream of services generated within that particular ecosystem (ecosystem service beneficiaries). Those responsible for providing ecosystem services usually have a title to use land and manage it in a way that maximizes the delivery of services demanded by the beneficiaries (e.g., representing upstream and downstream communities, respectively). The popularity of this approach has been growing with an increasing interest in market instruments for environmental conservation and with the growing adoption of the ecosystem services framework in environmental management.

Although PES are intended to solve environmental problems and at the same time alleviate poverty, they may actually aggravate the latter. We refer to such counterintuitive negative socio-economic consequences of PES as *ecosystem service curse* (Kronenberg and Hubacek 2013) and associate them principally with the problems of rent seeking, unequal bargaining power and volatility of payments, explained in the following three paragraphs.

Rent seeking may emerge when some actors take over payments (i.e., rents from ecosystem services), often by manipulation, corruption or force, from those who would have been entitled to receive those rents under normal circumstances. As PES are tied to land ownership, such problems may emerge when customary property rights are not strong enough to ensure that marginal local communities receive those payments or when these communities are driven off their land by powerful rent-seeking stakeholders. For example, such elite capture through the monopolization of access to forestland has been identified as one of key challenges to PES implementation in Vietnam. In particular, one case study—Ba Vi National Park—illustrates an extreme situation in which all payments were captured by local elites who used their connections to political power and thus monopolized access to land (To et al. 2012). Furthermore, when the indigenous communities are forced to move, they often encroach upon other usually more marginal areas, potentially moving them beyond a tipping point or otherwise contributing to their degradation.

Unequal bargaining power, i.e., the fact that ecosystem service beneficiaries have more experience with market mechanisms and negotiations, affects the conditions within which PES transactions are negotiated. Communities responsible for providing ecosystem services may have limited experience and understanding of such novel mechanisms, and few opportunities to seek competent and affordable legal advice. Some ecosystem service providers may even be excluded from the potential stream of benefits because of their inability to enter the relevant negotiations. Instead, payments may be directed to larger players who are easier to work with [indeed, for these reasons, the Costa Rican PES scheme has been critically assessed as an indirect state subsidy for large agribusiness (Lansing 2013)].

Volatility of payments may make it difficult to create viable long-term ecosystem management strategies. This volatility can be due to a number of factors beyond the control of the recipient. This can include changes to the respective ecosystems and their properties [often in response to external biophysical stressors, such as environmental degradation, forest fires, and sea level rise (Friess et al. 2015)], as well as changes in needs and preferences affecting how much people would be willing or are able to pay for ecosystem services. These translate into volatility of income for ecosystem service providers, which so far has been most evident in payments related to carbon sequestration due to price fluctuations in the international carbon market (Phelps et al. 2011).

Rent seeking, unequal bargaining power and volatility of economic circumstances contribute to the so-called poverty trap, i.e., the persistence of poverty, in spite of attempts to get out of it. Persistence of poverty is often explained by threshold effects (indicating that an individual, a group or even a country has to reach a certain level of wealth to be able to get out of poverty), dysfunctional institutions (that are not able to ensure proper means that would help people get out of poverty) and neighborhood effects (influences from one’s peers) (Bowles et al. 2006). Poverty traps can be linked to multiple barriers that prevent people from making their own choices and entering markets on equal terms (Sen 1993). Indeed, many attempts to provide environmentally sound development opportunities to poor communities in developing countries have not been able to break the poverty trap in which those communities are caught (Valkila 2009). We contend that the potential ecosystem service curse may represent yet another example of such problems and that these problems may gain additional relevance when PES schemes grow as predicted in the near future (Carroll and Jenkins 2008; Milder et al. 2010).

The ecosystem service curse hypothesis exceeds the socio-economic focus of most poverty trap discussions to cover social-ecological systems. Indeed, all of the above ecosystem service curse problems are related to the dynamics and complexity in social-ecological systems, and they ultimately depend on the quality of institutions that govern these systems. Institutions act as linkages between the social and the ecological, and require an understanding that these systems coevolve and mutually influence each other. Because of these linkages, sustainability science provides a useful lens to study the ecosystem service curse hypothesis, notably within the framework of social-ecological traps, as explained in the following sections.

## The ecosystem service curse and sustainability science

PES create new streams of capital, often flowing from more developed into less developed countries or regions endowed with valuable ecosystem services. Several problems have already been observed in the literature with regard to these payments and the negative effects that they might have on recipient communities. Many of these problems resemble those which had been identified within the case of the so-called resource curse hypothesis. This hypothesis indicates that resource revenues are highly correlated with economic problems in poor countries, which are not able to use those revenues to ensure sound development (van der Ploeg 2011). Indeed, our ecosystem services curse hypothesis suggests that once PES increase in scale they may assert a similarly negative influence in economies with rich endowments in ecosystem services. Such an increase in PES might lead to problems such as the exclusion of local communities from the land that they have been using or otherwise affecting their ability to benefit from such payments. Clearly, such problems so far have only emerged on a local scale and mostly affected poor ecosystem service providers, thus contradicting the often highlighted objective of PES, i.e., reducing poverty.

The above issues are frequently discussed in the context of sustainability—with regard to poverty and development disparities, and various challenges related to managing natural wealth. They refer to governance and to the needs of various stakeholders, including non-human species and future generations who are not sufficiently represented in the debate (Hubacek and Mauerhofer 2008). Indeed, they reflect interactions between global, social and ecological systems and the complex mechanisms that lead to their degradation, which are central to sustainability science. These issues extend beyond traditional disciplinary boundaries and require a broader social-ecological systems perspective—revealing problems associated with uncertainty and ignorance (regarding unintended consequences of new financial mechanisms), and demonstrate the need for a precautionary approach.

This is especially evident in the case of global financial mechanisms for mitigating climate change. Mechanisms such as Reducing Emissions from Deforestation and Forest Degradation in Developing Countries (REDD+) and the afforestation component within the Kyoto Protocol’s Clean Development Mechanism already use PES-like schemes on a global scale. The relevant institutional problems hindering the social and environmental effectiveness and economic efficiency of these programs provide good illustrations of a potential ecosystem service curse. For example, REDD+ payments, which are meant to reward practices that counteract deforestation and forest degradation and in this way prevent carbon emissions, mostly flow to countries with relatively poor institutions which are not able to counteract problems such as rent seeking or payment volatility (Ebeling and Yasué 2008; Kronenberg et al. 2015; McAfee 2015).

Similar problems have been observed in the case of other newly created markets for ecosystem services. These tend to turn ecosystem services into cash crops (grown for sale to return a profit) and can lead to the abandonment of traditional land management and local cultural practices (e.g., eroding traditional value systems), and exposing ecosystem-dependent communities to unfavorable power structures and economic dynamics (Sikor 2013; Pröpper 2015; Van Hecken et al. 2015). Among the most notorious manifestations of these are the so-called land grabs within which powerful stakeholders exert their high bargaining power to enforce new land use schemes—to the detriment of smallholders and especially land users who are not protected by proper land tenure systems. As part of these large-scale land acquisitions or long-term leases in poorer countries land use and land management decisions are transferred to foreign powerful stakeholders. Indeed, these have often been associated with exploitation by corrupt or indebted governments that were not able or willing to properly regulate these transactions or prevent beneficiaries from targeting and taking advantage of the poorest rural communities (Deininger and Byerlee 2011; Cotula 2012). These problems have also emerged in the case of several PES projects, including the famous Pimampiro case in Ecuador which has been found to reinforce existing social differences and perpetuate inequalities in resource access, indicating that those with higher bargaining power have been able to attract most funding (Rodríguez de Francisco et al. 2013),

Ecosystem services and the related financial mechanisms are expected to constitute a proper framework for addressing the problems that sustainability science deals with (Clark 2007). However, on an even broader level, problems depicted within the ecosystem service curse hypothesis reveal more general complications with the concept of ecosystem services and the application of economic solutions to sustainability challenges. Indeed, economic concepts do not neatly fit into sustainability science (Anderson et al. 2015; Wegner and Pascual 2011) and their underlying utilitarian and anthropocentric focus trivializes the complex character of social-ecological systems, thus broadening the scope of unexpected outcomes. In particular, introducing economic incentives results in profound changes within the socio-economic sphere, releasing human ingenuity to capture these incentives at potential long-term costs (Kronenberg 2014, 2015).

## The ecosystem service curse as a social-ecological trap

**Fig. 1:**
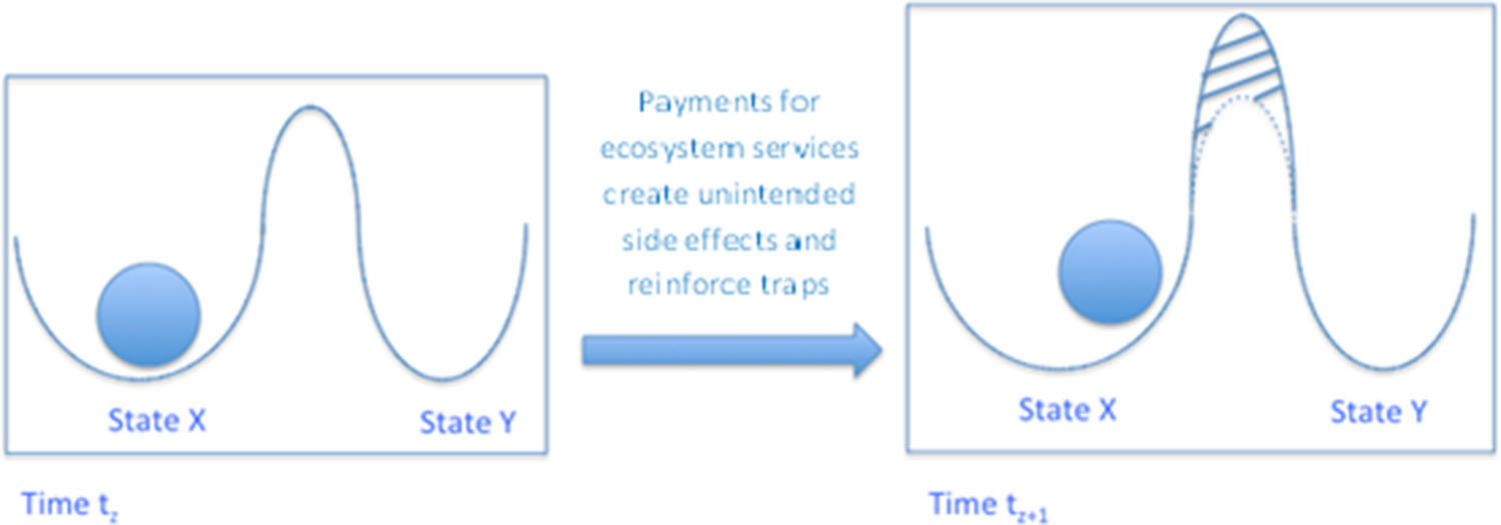
Within an ecosystem service curse the trap situation is reinforced and the system is forced to stay within its basin of attraction (the *x*-axis presents different stability domains)

From the perspective of social-ecological systems theory, the concept of an ecosystem service curse illustrates a situation where an alteration of social and ecological processes leads to livelihood impoverishment and sometimes even fails to achieve environmental improvement or, worse, brings about additional environmental degradation. In this case, the unintended side effects of introducing a payment reinforce the trap situation (its basin of attraction) and an initial intervention aimed at improving ecosystem management produces counterintuitive socio-economic consequences (Fig. 1).

As suggested by Boonstra and Boer (2014), “social–ecological traps are clearly path-dependent processes, which are causally produced through a conjunction of events”—thus traps are dynamic processes rather than static conditions. Although we focus on poor institutional setting that is the precondition of an ecosystem service curse, it is the process of introducing PES and the reaction of the system that ultimately reinforce the initial trap situation, which this intervention was meant to solve. Karsenty (2007), who referred to a similar problem with PES, suggested that PES might keep poor communities in a poverty trap when they receive payments for refraining from activities that might negatively affect the ecosystem’s capacity to provide hitherto unpaid services. Following his interpretation of the poverty trap, these communities might become passive “conservation rentiers”, losing any dynamism and innovation potential they might have had, had they pursued their traditional development path or an alternative one. Our view of a curse emphasizes that additional factors need to be taken into consideration when designing PES, such as the likelihood of excluding poor and ecosystem-dependent communities from “their land” through rent seeking or unfavorable contract formulation exploiting their low bargaining power.

The ecosystem service curse links to both poverty traps and rigidity traps, with the former associated with people “impoverished by circumstances beyond their control” and not realizing the potential for change, and the latter focused on inflexible institutions and corruption (Carpenter and Brock 2008). Interestingly, the ecosystem service curse may be a side effect of excessively emphasizing economic thinking as a way to solve complex social-ecological problems (Norgaard 2013). As a result, society keeps being surprised with unintended and counterproductive consequences of its activities (Faber et al. 1992). In an ecosystem service curse situation there are not only unintended side effects further disadvantaging some social agents but there are also very important interests and winners involved, which makes reaching the initially intended outcome by the PES even more difficult. There might be rentiers with large return on investment achieving even an increase in the delivery of the focal ecosystem service (but at the abovementioned social and potentially also ecological cost—affecting poor communities and other ecosystems). Poor communities that depend on ecosystems for their livelihoods may either be excluded from using their land or be subordinated to the needs of external stakeholders (ecosystem service beneficiaries). In this way they risk not only losing opportunities to benefit from PES, but also losing their traditional sources of income. This may result in additional degradation of ecosystem services that are deemed less valuable in a given market situation.

Thus, the ecosystem service curse exhibits the intertwined problems of complexity and ignorance. Within sustainability science we need to follow systems thinking, taking dynamics into account, and acknowledging complexity (Holling 2001; Cilliers 2008). It is not only the ecological complexity that we need to take into account but a social-ecological complexity, within which the social component is also highly variable. Analogous to the “out of the frying pan, into the fire” trap, when actions intended to escape a trap accidentally worsen the situation, the introduction of PES may lead to negative social and ecological consequences. A precautionary approach suggests that we should consider potential problems already at the PES design stage (if one is compelled to introduce a PES scheme), taking into consideration the likelihood of surprises given the messiness of the institutional and social context as well as the volatility of markets and preferences.
